# Comparative Investigation of the Experimental Determination of AA5086 FLCs under Different Necking Criteria

**DOI:** 10.3390/ma14133685

**Published:** 2021-07-01

**Authors:** Xiangrui Kong, Xingrong Chu, Chongqian Chen, Yangang Wang, Peixing Liu, Zhihao Wang

**Affiliations:** 1 Associated Engineering Research Center of Mechanics and Mechatronic Equipment, Shandong University, Weihai 264209, China; kongxr1367@sina.com (X.K.); zhihaow_edu@163.com (Z.W.); 2 Tianjin Jiurong Industry Tech Co., Ltd., Tianjin 300380, China; chenchongqian@126.com; 3 Iron and Steel Group Rizhao Co., Ltd., Rizhao 276800, China; qunxing985@126.com

**Keywords:** forming limit diagram, necking criteria, Marciniak test, Nakajima test

## Abstract

The construction of a forming limit diagram (FLD) is a conventional approach to obtain limit strains and to evaluate the formability of sheet metal. Appropriate necking criteria should be applied to determine the forming limit curve (FLC) accurately. In recent years, deep research on the determination of the FLC has been carried out; meanwhile, several necking criteria have been proposed. However, the application of inappropriate necking criteria would cause deviations when determining FLCs. In this study, both Marciniak and Nakajima tests were carried out on the AA5086 aluminum sheet to make a comparative investigation of different necking criteria in the determination of FLCs. In the Marciniak test, four existing necking criteria were chosen to construct FLCs, and analyzed in detail. The well-performed time dependent and position dependent methods were selected for the Nakajima test. Meanwhile, the modified Wang method based on the height change of the adjacent points was proposed. The comparative results showed that the time and position dependent methods were relatively conservative in both experiments, while the modified Wang method could identify the onset of localized necking more accurately.

## 1. Introduction

Metal sheet forming has been widely used in various industrial production processes, especially in the automotive [1,2] and aeronautic [3] fields. A lightweight design based on metal sheet forming has become popular in industrial production. However, the metal formability has generally been restricted by the failure limit corresponding to the onset of localized necking. The forming limit diagram (FLD) raised by Keeler and Backofen [4] has been a common method for the evaluation of metal formability. By measuring the limit deformation of the metal sheet under different strain paths, the forming limit curve (FLC) could be constructed according to the critical major and minor strains. Afterwards, safety and failure regions are separated by the shape and location of the FLC.

Precise construction of the FLC has been regarded as taskwork. Commonly used methods to determine the FLC have included the theoretical method, finite element (FE) simulation, the experimental test, etc. The theoretical method, requiring complex mathematical analysis, might cause deviations due to the approximation of empirical equations or the variation of certain constant values (e.g., the hardening index considered as a constant might change with material deformation [5]). Fundamentally, the main limitation in the analytical models, e.g., the MK-framework, has lied in the need for a prior forming limit curve commonly utilized to calibrate the analytical model [6]. It has been more convenient to obtain the FLD through numerical simulation. However, predicted limit strains might be inconsistent with the actual forming limits due to the deviation of the mesh division or friction coefficient selection, etc. According to Zhang et al. [7], though the FE methods evaluates the metal formability effectively, their agreement with experimental results was not perfect; Heidari et al. [8] also found that the simulation results showed a large deviation on the right side of the FLD when they utilized the ductile fracture criteria to assess the formability of AA6063 alloy. Therefore, the results of numerical simulations still need to be validated by experimental tests. Among these test methods, the out-of-plane Nakajima test [9] and in-plane Marciniak test [10] are the most prominent experiments. In the Nakajima test, the specimen is deformed under a hemispherical punch and subjected to triaxial stress; meanwhile, the through-thickness stress in the Marciniak test is small enough to be neglected. Therefore, specimen in the Marciniak test can be considered as plane stress conditions in the central part. Marrapu et al. [11] utilized both numerical and experimental methods to assess the formability of DP780 steel; the consequences showed that the simulation based on the major true strain gradient method (strain localization criterion in [12]) had great consistency with the experimental results. Likewise, Nakajima tests on DC04 steel were developed by Lumelskyj et al. [13] to validate the accuracy of numerical results under two different necking criteria. The thinning rate evolution criterion gave the values of limit strains closest to the experimental ones, while the maximum strain acceleration criterion overestimated the limit strains compared to the experimental ISO norm FLC. It seems, therefore, that the development of experiments is still the most reliable and practical method to determine the FLD.

Appropriate necking criteria should be selected to define the onset of localized necking, which is considered an essential issue in precisely determining the FLD. Necking criteria mainly contain two categories: the position dependent method and the time dependent method. A clear standard of the position dependent method has been provided in ISO 12004 (2008) [14], while the time dependent method proposed by Eberle et al. [15] is still under standardized. In recent years, researchers have spent great efforts analyzing the effect of different criteria on the determination of the FLC: different temporal and spatial criteria were compared by Wang et al. [16] in the Marciniak test. The results showed that spatial methods determining the area after fracture presented larger limit strains, whereas temporal methods determining the diffuse necking area led to smaller consequences. Meanwhile, a new method based on monitoring the surface topography of specimens has been proposed, which is considered as the most accurate method for measuring the boundary of the safety area. S Dicecco et al. [17] tested a necking zone (NZ) method proposed by Martínez-Donaire et al. [18] and found that the limit strains obtained by ISO 12004-2 and the NZ method at room temperature were almost identical on the uniaxial of the FLC, while the NZ approach yielded a 14.8% larger major limit strain on the biaxial side. A time-position-dependent method (flat-valley method) based on the appearance and development of a valley in the profile was also demonstrated in [18]. The initiation of necking was inferred when the slope in the first spatial derivative of the vertical displacement remained constant within the necking region. Based on surface geometry measurement, an improved 3D curvature method was proposed by Min et al. [19] from their previous 2D curvature works [20]. The FLC could be obtained by this method on an equivalent basis from both Marciniak and Nakajima tests after applying compensation for the effects of the non-linear strain path, curvature and contact pressure.

Chalal and Abed-Meraim [21], who applied four necking criteria in the Nakajima test, verified that numerical simulation FLDs predicted by local criteria were in good agreement with the experimental results. By contrast, the global criterion based on the maximum punch force on the left side of the FLD seemed not to be suitable for the prediction of local necking. A method based on thickness variation was proposed by Iquilio et al. [22] and compared with two existing criteria. This thickness variation correlation method was proved to be more accurate to determine the true limit strain. Lumelskyj et al. [13] compared two different time dependent methods with the ISO standard. The through-thickness strain method proposed by Volk and Hora [23] was defined by the point corresponding to a sudden change to the slope of the thinning rate versus the time curve, which showed quite similar results to ISO 12004-2. Further, the maximum of the strain acceleration method, which obtained the strain localization by determining the inflection point in the major strain rate curve, gave higher values of strains than ISO. 

Although plenty of failure criteria and correlative optimization tests have been introduced, comparisons of the characteristics and applicability of different necking criteria are still needed, especially through the experimental way. Therefore, in this paper, both Marciniak and Nakajima tests were used to construct FLCs of the AA5086 aluminum sheet. In the Marciniak test, four different failure criteria were applied to determine the FLCs, and the results were compared to assess the applicability of different criteria in the formability evaluation. In the Nakajima test, the well-performed time and position dependent methods of the Marciniak test were selected to determine FLCs. Meanwhile, a modified method based on [16] was proposed and tested as well. Finally, the FLCs predicted by three different criteria were compared and the applicability of the modified method was briefly described.

## 2. Experiments and Discussion

- **Part I Marciniak**

### 2.1. Experimental Apparatus

**Figure 1:**
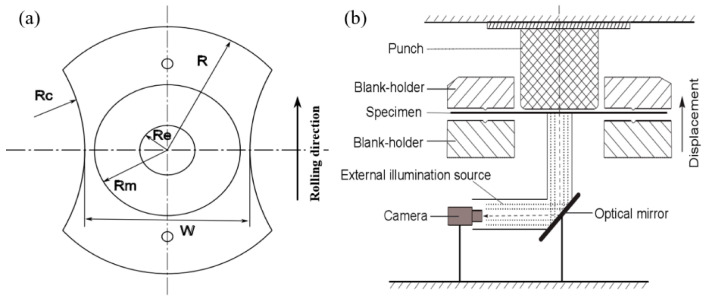
Experimental equipment and specimen: (**a**) Specimen geometry, (**b**) Schematic of the Marciniak acquisition system.

A standard Marciniak apparatus was revised to investigate the forming limit of AA5086, with a punch diameter of 40 mm and a die diameter of 43.8 mm. The punch corner radius was 8 mm and the die corner radius was set to 5 mm. A serrated surface was designed on the die to better grip the specimen. To assure the occurrence of maximal strains on the central part of the blank, and to also void the friction between the blank and the punch, the sheet adopted in this study was machined with a non-uniform thickness, as presented in Figure 1a. The central zone (Re) had a thickness of 0.8 mm, the adjacent zone (Rm), 1.5 mm, and the clamping zone, (R) 2.0 mm. Related information about the specimen dimensions is shown in Table 1. The widths of the specimen were changed from 10 to 100 mm in order to obtain different strain paths. The other radius dimensions remained the same for the different strain paths. Each test was carried out at least three times.

The digital image correlation (DIC) method was used to capture the images of samples during testing. The schematic diagram of the experimental acquisition apparatus is presented in Figure 1b. The CMOS camera with a resolution of 512 × 448 pixels and a shutter speed of 500 images/s was adopted. A subset size of 32 pixels and a step size of 16 pixels were utilized for analysis. All specimens were covered in white paint and then sprayed with black dots on their surface to analyze the deformation by means of the captured images.

### 2.2. Different Criteria to Determine the FLC

#### 2.2.1. Position Dependent Method

This criterion was explicitly explained in ISO 12004-2. This spatial method is based on the strain distributions in the specimen prior to the appearance of fractures. The theory of the ISO standard is that, with a fit window of the chosen main strain values ($\varepsilon_{11}$, $\varepsilon_{22}$) on both sides of the necking zone for a necking prior to a cracked specimen, an inverse polynomial function of second-order Equation (1) is fitted to identify the limit strains at the start of necking:

(1) $$f\left(x\right)=1/\;\left(ax^{2}+bx+c\right)$$

**Figure 2:**
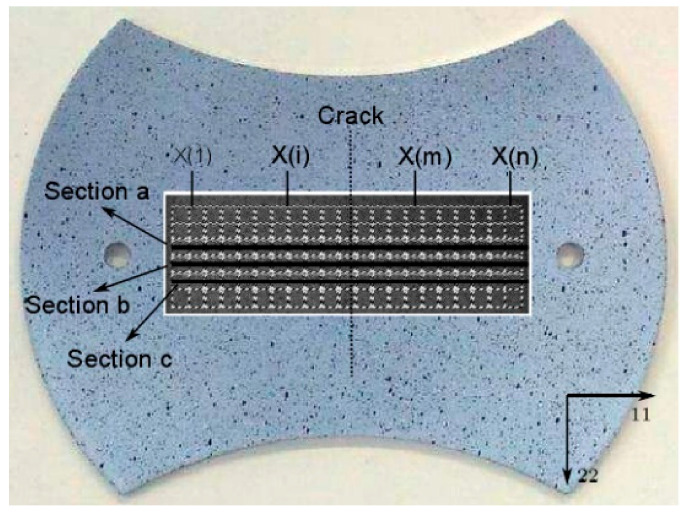
Locations of the three relevant cross sections.

**Figure 3:**
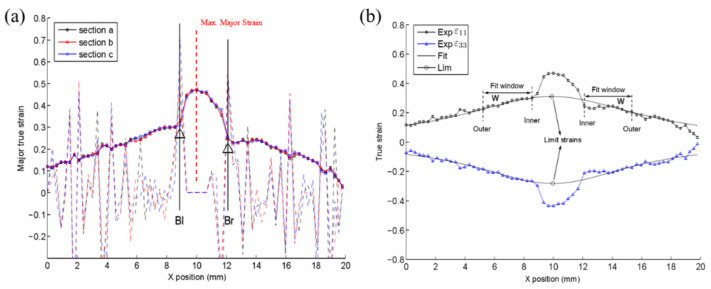
(**a**) Determination of the inner boundary of the fit window, (**b**) Limit strains determined by inverse fit parabola.

To allow a reproducible evaluation, three related cross sections, including the crack, were selected to investigate the strain distribution, as shown in Figure 2. The X(m) represents the position values of each section point on the specimen surface, which were obtained by the DIC method. With correlative data, the major strain distributions of the chosen sections on the sheet metal before the occurrence of a crack could be obtained, and a procedure of a second derivative was applied on these strain evolutions. The inner boundary of the fit window was determined by the maximum value point of the second derivative at each side of the crack, as presented in Figure 3a. The dashed lines presented second derivatives of the points at both sides.

To find the outer boundary of the fit window, ISO 12004-2 defined the fit window width *W* for each side in Equation (2):

(2) $$\begin{array}{c} W=4\left[1+\left({\bar{\varepsilon }}_{22}/\;{\bar{\varepsilon }}_{11}\right)\right]\\ {\bar{\varepsilon }}_{22}=\frac{1}{2}\left(\varepsilon_{22,Bl}+\varepsilon_{22,Br}\right)\\ {\bar{\varepsilon }}_{11}=\frac{1}{2}\left(\varepsilon_{11,Bl}+\varepsilon_{11,Br}\right) \end{array}$$

where ($\varepsilon_{11}$, $\varepsilon_{22}$) are the principal strain values of the inner point at each side, *Bl* stands for the left inner boundary and *Br* stands for the right inner boundary.

It was found that the determined strain path ($\beta_{exp}=\varepsilon_{11,\mathrm{limit}}/\varepsilon_{22,\mathrm{limit}}$) did not always correspond to the measured strain path ($\beta_{exp}$), which was measured directly by the DIC. Therefore, the limit strain $\varepsilon_{22,\mathrm{limit}}$ in this test was directly determined by the ($\beta_{exp}$) in order to limit data scatter, with the relation ($\varepsilon_{22,\mathrm{limit}}=\beta_{exp}\cdot \varepsilon_{11,\mathrm{limit}}$). The thickness true strain ($\varepsilon_{33}=-\varepsilon_{11}-\varepsilon_{22}$) was obtained based upon the constancy of volume. Utilizing the inverse best-fit parabola (Equation (1)) of the strain points over the determined fit window, the function’s maximum peak was identified as the limit strains ($\varepsilon_{11,\mathrm{limit}},\varepsilon_{33,\mathrm{limit}}$), as illustrated in Figure 3b.

#### 2.2.2. Time Dependent Method

In ISO 12004-2 norm, a “time dependent” method was introduced to be still under development. A termed time dependent evaluation criterion was presented by Merklein et al. [24] in the literature. This criterion was performed to analyze a tendency of the strain rate in the zone of necking and the succeeding cracking. By means of the image prior to the crack occurrence, the distribution of the major strain was acquired, as formerly presented in Figure 3a. The maximum major strain point was selected to begin the assessment and an average value of the three sections was adopted to obtain stable measurement results.

**Figure 4:**
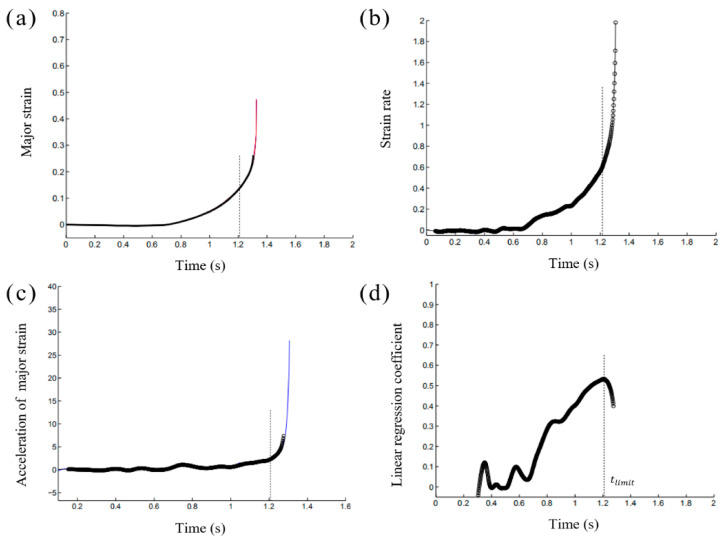
Time dependent method: (**a**) Evolution of major strain, (**b**) Evolution of major strain rate, (**c**) Acceleration of major strain, (**d**) Linear regression coefficient.

The different processes of this temporal criterion are presented in Figure 4. Both major strain and major strain rates presented a homogeneous development at the initial deformation period and then both values increased drastically at the onset of necking. The acceleration of major strain developed linearly at the start of deformation (Figure 4c), followed by a rapid rise. Then, a linear regression coefficient of the major strain acceleration was obtained with calculations. The values of the linear regression coefficient started to increase along with the continuous homogeneous plastic deformation, achieving a maximum peak at the onset of necking. After the occurrence of necking, the major strain acceleration showed a drastic increase, while the linear regression coefficient declined. The maximum peak in the linear regression coefficient’s evolution indicated the instant for the onset of necking (Figure 4d), and the relevant limit strains at this moment were noted as one point on the forming limit curve.

#### 2.2.3. Strain Increment Ratio Criterion

**Figure 5:**
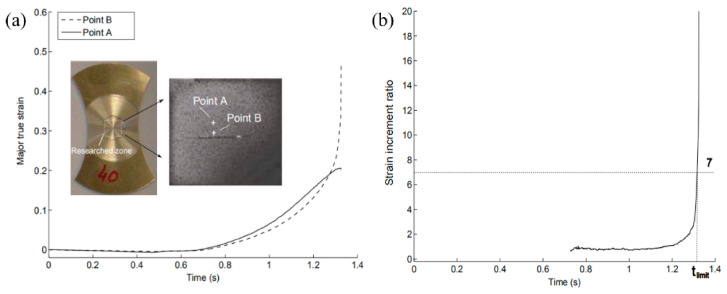
(**a**) Major true strain evolution of different zones, (**b**) Strain increment ratio criterion.

This method is frequently adopted in the M-K theory [10] (the most widely used theoretical model in which an initial geometrical imperfection is assumed to trigger the occurrence of the localized necking), on account of its simplistic definition. This temporal method is based on the strain evolutions’ difference between necking and the adjacent areas. Two points (point B in the necking area and point A in the adjacent area) on the sample surface were selected. After strain localization, the strain discrepancy between the two areas sped up, as presented in Figure 5a. As the major true strain increment ratio (${\Delta \varepsilon }_{\mathrm{Major}}^{B}/{\Delta \varepsilon }_{\mathrm{Major}}^{A}$) transcended the critical value (7 was proposed by C. Zhang [25]), necking was supposed to take place, and the corresponding limit strains of point B at this instant were regarded as one point of the forming limit curve, as shown in Figure 5b.

Nonetheless, with this criterion, the limit strain values could be affected by the positions of points A and B and the interval time value utilized in the strain increment ratio calculation. For the same investigated points, the major and minor strains measured with different time intervals are presented in Table 2. As the interval time increased, a slight increase of the forming limit value could be observed. The major and minor strains obtained with a Δt of 0.01 s displayed the most conservative consequence. This interval time Δt = 0.01 s remained in the subsequent comparisons.

#### 2.2.4. Maximum Punch Force Criterion

**Figure 6:**
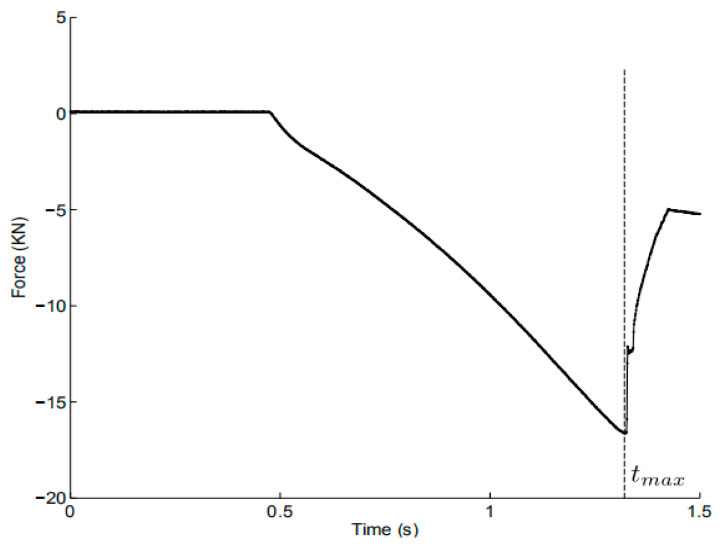
Evolution of recorded punch force in the Marciniak test.

The punch force was recorded by a traditional load cell across the duration of the Marciniak test, as presented in Figure 6. Meanwhile, the deformation of the specimen was observed through the high-speed camera. The punch force presented a drastic increase until reaching the maximum point, and suddenly decreased as soon as the sheet failure occurred. Therefore, the force evolution is feasible to be utilized as a global criterion to determine the limit strains. The three sections adopted in the ISO standard were selected. The maximum strains in the chosen sections with respect to the maximum force instant were obtained with strains calculated by the DIC system. The mean values of the three maximum strains were recorded as the limit strains.

### 2.3. Results

**Figure 7:**
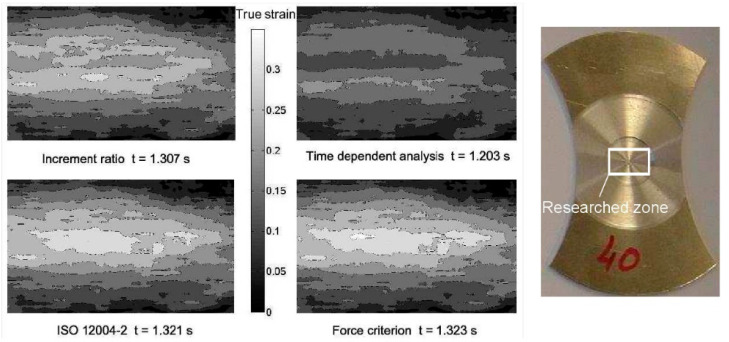
Mises effective strain distribution of the specimen corresponding to different critical times.

For the investigated sheet metal with a width of 40 mm, the limit strains and corresponding times determined with the various necking methods are shown in Table 3. The Mises effective strain distributions in the necking area corresponding to the different critical instants were determined by the CORRELA 2006 software, as presented in Figure 7. The smallest determined instant was given by the time dependent method, while the largest was obtained with the force method. Additionally, the strain localization obtained with the time dependent criterion was not clear, but the onset of necking could be clearly observed from the results of the force criterion.

**Figure 8:**
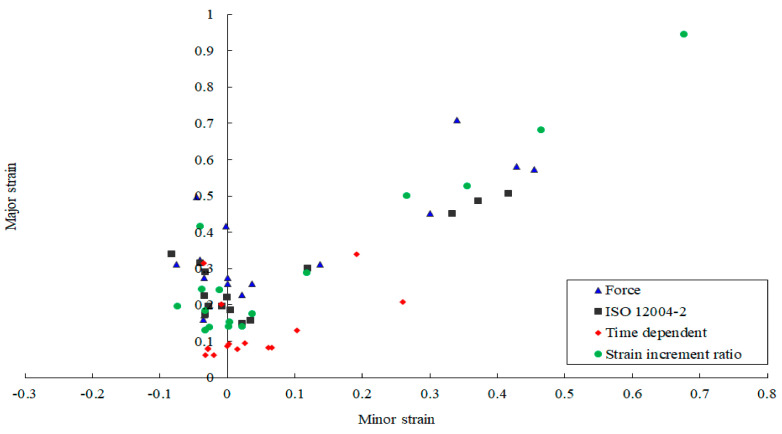
The FLCs of the Marciniak test with different failure criteria.

The FLCs of the given specimen AA5086 determined with the various necking criteria are presented in Figure 8. The results indicated that the limit strains distinctly relied on the selection of the necking criteria. The position dependent method gave the most regular and repeatable data, which proves its robustness. It has been standardized and recognized as a reliable failure criterion with wide application in the industry. However, based on the assumption of the centrally fitted parabola, this method revealed a relatively rigorous requirement on the location of cracks. If the crack deviated from the center location, it might be the significant asymmetry in strain measuring which led to the inaccurate parabola fitting results.

The time dependent criterion gave very conservative results. This method was well applicable to materials with a distinctive necking period before the occurrence of the crack. As for the tested AA5086 specimen, this method might determine the diffuse necking rather than the localized necking, and consequently, conservative FLC results were obtained. 

Dispersed data were obtained with the strain increment ratio criterion in the uniaxial and plane strain areas. For this method, only one point in the necking area was selected. The choices of the point position, interval time Δt and the strain increment critical value all affected the limit strain values. 

The maximum force method presented scattered data results for some samples. For the duration of the Marciniak test, the punch force was affected by many factors, such as the lubrication conditions. As a result, the maximum force method seemed to be unsuitable for determining the limit strains.

- **Part** **Ⅱ Nakajima**

### 2.4. Experimental Apparatus

**Figure 9:**
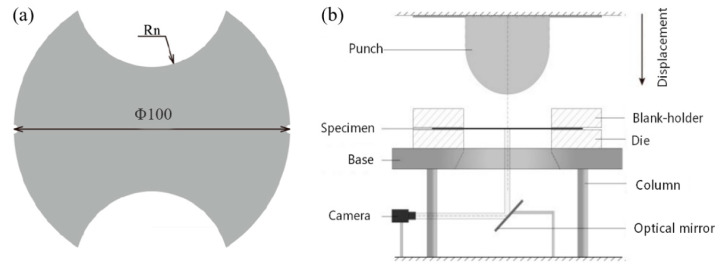
(**a**) Specimen’s geometry in the Nakajima test, (**b**) Schematic of the Nakajima acquisition system.

A Nakajima test model was installed on a universal mechanical testing machine. The punch with a diameter of 44.45 mm moved down the die (49 mm diameter) to stretch the AA5086 specimen. The deep drawing ribs owned a radius of 6 mm, and were symmetrically located on the blank-holder with a diameter of 79 mm. The specimen’s geometry is shown in Figure 9a. In order to construct the FLD with complete strain paths, seven specimens with different sizes were obtained by changing the width of the geometry. Detailed dimensions of the specimen are shown in Table 4. Grooves were provided on both sides of the specimen dome to facilitate positioning during the test. During the test, the specimen was clamped between the die and the blank holder by bolt fastening. The deep drawing ribs on the die and blank holder ensured the pressed part of the material would not flow during the stamping process. Each test was carried out at least three times.

To record the specimen image during the deformation, two CCD cameras with the solution of 1624 × 1236 pixels were used, and the acquisition of 2 images/s was adopted. In this study, a subset size of 7 pixels and a step size of 14 pixels were chosen for the correlation analysis. A speckle image of the specimen surface during deformation was recorded by the camera and was used to calculate the strain field. The schematic diagram of the analysis system is shown in Figure 9b.

### 2.5. Different Criteria to Determine FLC

It could be seen from the results of the previous Marciniak test that position dependent and time dependent criteria gave more concentrated (not scattered) limit strain points and performed well in the evaluation of the formability of AA5086 sheet. Therefore, in this work, these two criteria were retained in the Nakajima test and compared with a modified method. Different FLC results determined by these three necking criteria are compared and discussed at the end of this section.

#### Modified Wang’s Method

**Figure 10:**
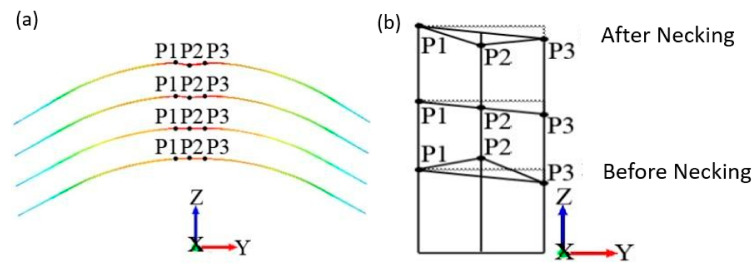
Variation of adjacent points in the high strain zone: (**a**) Cloud image, (**b**) Geometric calculation diagram.

A modified necking criterion based on the height change of adjacent points in the necking area was proposed in this work. It had been modified to be feasible for the Nakajima test based on the work of Wang et al. [16]. During the forming process, the DIC system was utilized to record and analyze the changes in surface topography (thickness changes). The variation of the thickness strain before and after necking is shown in Figure 10a. With the increment of deformation, the position of maximum strain gradually transformed from convex to concave. The point P2 was at the maximum strain zone, and P1 and P3 were its adjacent points. Before necking, P2 was higher than the line between P1 and P3. As the specimen deformation increased, the height of P2 gradually changed from higher than the line (P1–P3) to parallel with it. Finally, the position of P2 became lower than the line (P1–P3). The geometric calculation diagram based on the above analysis is drawn in Figure 10b. P1, P2, and P3 satisfied the following geometrical relationship:

(3) $$\{\begin{array}{c} z_{2}>\frac{z_{1}-z_{3}}{y_{1}-y_{3}}y_{2}+z_{3}-\frac{z_{1}-z_{3}}{y_{1}-y_{3}}y_{1}\;Before\;necking\;\\ z_{2}<\frac{z_{1}-z_{3}}{y_{1}-y_{3}}y_{2}+z_{3}-\frac{z_{1}-z_{3}}{y_{1}-y_{3}}y_{1}\;After\;necking \end{array}\;$$

*y* and *z* are the coordinates of each point, and ($\frac{z_{1}-z_{3}}{y_{1}-y_{3}}y_{2}+z_{3}-\frac{z_{1}-z_{3}}{y_{1}-y_{3}}y_{1}$) is the intersection of the line (P1–P3) and the vertical line of P2.

Let

(4) $$\Delta z=\frac{z_{1}-z_{3}}{y_{1}-y_{3}}y_{2}+z_{3}-\frac{z_{1}-z_{3}}{y_{1}-y_{3}}y_{1}-Z_{2}$$

**Figure 11:**
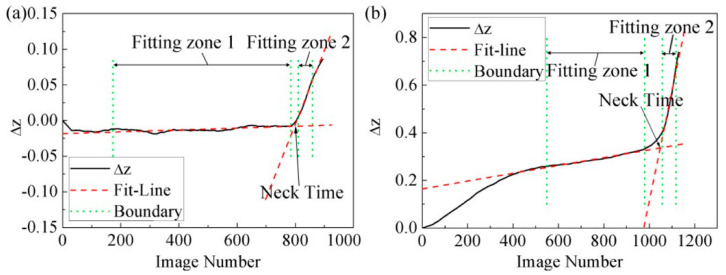
Variation tendency of  Δz and necking time: (**a**) Uniaxial stretching strain region, (**b**) Biaxial stretching strain region.

The tendency of  Δz changing with time is shown in Figure 11. In the entire deformation region,  Δz displayed a linear variation period at the beginning (Fitting Zone 1), and then increased rapidly (Fitting Zone 2). The onset of localized necking was perceived as occurring on the process of transforming from Fitting Zone 1 to Fitting Zone 2. Based on this, linear fitting was performed, respectively, on Fitting Zone 1 and Fitting Zone 2. The abscissa at the intersection of two straight lines was defined as the initial necking time, and the limit strain at this time was obtained by linear interpolation between the two measured adjacent strain points. 

### 2.6. Results

**Figure 12:**
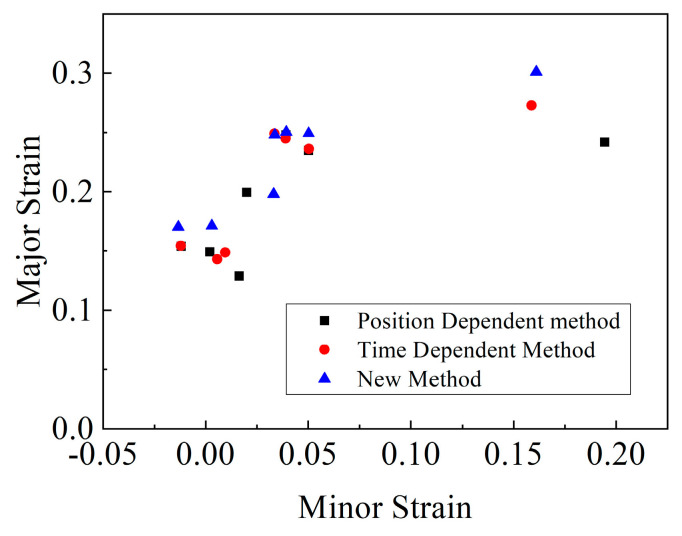
Comparison of FLCs in the Nakajima test under different necking criteria.

For the three different necking criteria mentioned above, the left side of the lowest point was fitted linearly and the right side was fitted by the exponential decay function. The obtained experimental FLCs are shown in Figure 12. The results indicated that the time and position dependent method were quite similar on the left side of the FLCs, while a slightly higher limit strain was obtained by the time criterion, rather than the position dependent method, on the right side. Further, the FLC defined by the modified method generally exceeded the other FLCs. Overall, the position dependent method seemed more conservative than other criteria for the construction of FLCs.

The modified Wang method was used to measure the thickness variation of the specimen’s surface. The onset of localized necking could be defined when the Δz changed sharply. Compared with the time dependent method, which measured the diffuse necking to determine the FLC, the modified Wang method obtained larger critical limit strains which seemed to be closer to the onset of localized necking. Moreover, based on the physical deformation of the metal sheet, this method directly displayed the failure of the materials, and only a simple post-processing procedure was needed. As for materials with large and uniform deformation, only linear interpolation was required to determine the initial moment of necking. However, for materials with a short time from necking to fracture, the changes of Δz could be very small and deviation might have been produced by applying this method.

## 3. Conclusions

In this paper, experimental Marciniak and Nakajima tests were used to study the effect of necking criteria on the determination of the FLCs for AA5086 aluminum sheet. Different necking criteria were applied to obtain limit strains, and the results were compared and discussed in order to make an evaluation of their applicability. The conclusions were drawn as follows:

For both the Marciniak and the Nakajima tests, the position dependent method based on ISO 12004 gave quite conservative results. Whether on the uniaxial or biaxial stretching side, the data given by this method were reliable, with good repeatability. 

In the Marciniak test, the time dependent method gave the most conservative limit strain data. In the Nakajima test, the time dependent method gave quite similar results as the position dependent method on the left side of the FLCs, while slightly higher limit strains were obtained with the time dependent method on the right side.

The strain increment ratio method predicted the onset of localized necking by comparing the ratio of strain in the necking and non-necking areas. Due to the fact that only one reference point in the necking zone was selected, this method might not perfectly reflect the general variation of the necking zone.

The limit strain points obtained by the global criterion maximum punch force were dispersed. The maximum punch force method was affected by many factors and hence seemed not to be suitable for determining limit strains.

The modified Wang method measured the changes to the specimen’s surface before and after necking to determine the onset of localized necking, and this gave higher values of limit strain than the time and position dependent criteria for the Nakajima test. For materials that possessed a short time from initial necking to fracture, the variation of Δz was hard to observe; thus, some deviation might have occurred when using this method.

## Figures and Tables

**Table 1:** Dimensions of the specimen during different strain paths.

**W (mm)**	10	20	30	40	45	48	50	52	55	58	60	80	100
**R (mm)**		**Rc (mm)**				**Rm (mm)**				**Re (mm)**			
50		70				26.5				10			

**Table 2:** Different interval times with corresponding forming limit values.

Interval Time (s)	t Limit (s)	Major Strain	Minor Strain
0.01	1.307	0.2448	−0.0385
0.02	1.309	0.2566	−0.0395
0.03	1.312	0.2682	−0.0404
0.04	1.316	0.2856	−0.0405

**Table 3:** Major and minor strains and corresponding times with different methods.

Methods	t (s)	Major Strain	Minor Strain
Strain increment ratio	1.307	0.2448	−0.0385
ISO 12004-2	1.321	0.3149	−0.035
Time dependent	1.203	0.1344	−0.0293
Maximum Force	1.323	0.3174	−0.0405

**Table 4:** Dimensions of the specimen’s geometry in the Nakajima test.

**R (mm)**	100						
**Rn (mm)**	0	16.7	19.4	22.2	33.3	38.9	44.4

## Data Availability

The processed data required to reproduce these findings cannot be shared at this time as the data also forms part of an ongoing study.
